# A microscopic landscape of the invasive breast cancer genome

**DOI:** 10.1038/srep27545

**Published:** 2016-06-10

**Authors:** Zheng Ping, Yuchao Xia, Tiansheng Shen, Vishwas Parekh, Gene P. Siegal, Isam-Eldin Eltoum, Jianbo He, Dongquan Chen, Minghua Deng, Ruibin Xi, Dejun Shen

**Affiliations:** 1 Department of Pathology, Division of Anatomic Pathology,University of Alabama at Birmingham, Birmingham, Alabama 35249, USA.; 2 Comprehensive Cancer Center, University of Alabama at Birmingham, Birmingham, Alabama 35249, USA; 3 Department of Medicine, Division of Hematology/Oncology, University of Alabama at Birmingham, Birmingham, Alabama 35249, USA; 4 Division of Preventive Medicine, University of Alabama at Birmingham, Birmingham, Alabama 35249, USA; 5 Center for Quantitative Biology, Peking University, Beijing 100871, China; 6 School of Mathematical Sciences and Center for Statistical Science, Peking University, Beijing 100871, China

## Abstract

Histologic grade is one of the most important microscopic features used to predict the prognosis of invasive breast cancer and may serve as a marker for studying cancer driving genomic abnormalities *in vivo*. We analyzed whole genome sequencing data from 680 cases of TCGA invasive ductal carcinomas of the breast and correlated them to corresponding pathology information. Ten genetic abnormalities were found to be statistically associated with histologic grade, including three most prevalent cancer driver events, TP53 and PIK3CA mutations and MYC amplification. A distinct genetic interaction among these genomic abnormalities was revealed as measured by the histologic grading score. While TP53 mutation and MYC amplification were synergistic in promoting tumor progression, PIK3CA mutation was found to have alleviated the oncogenic effect of either the TP53 mutation or MYC amplification, and was associated with a significant reduction in mitotic activity in TP53 mutated and/or MYC amplified breast cancer. Furthermore, we discovered that different types of genetic abnormalities (mutation versus amplification) within the same cancer driver gene (PIK3CA or GATA3) were associated with opposite histologic changes in invasive breast cancer. In conclusion, our study suggests that histologic grade may serve as a biomarker to define cancer driving genetic events *in vivo*.

Whole genome sequencing technology has led to a paradigm shift in the way that researchers view, study and understand breast cancer biology. To date, multiple whole genome breast cancer sequencing projects have yielded enormous data that have greatly facilitated our understanding of breast cancer initiation and progression, with the hope of eventually improving clinical patient management^1^,^2^,^3^,^4^,^5^,^6^,^7^. Much of these data have now been deposited in The Cancer Genome Atlas (TCGA) site (http://cancergenome.nih.gov/) and are publicly available to the scientific community. As TCGA project is approaching its completion, an effort has now been made to prioritize the TCGA data analysis to better understand cancer development and progression and to translate the knowledge into personalized or precision oncology^8^. One of the major goals of the cancer genome sequencing project was to identify all cancer driver genes, which were defined as those genetic abnormalities that conferred upon tumor cells selective growth or clonal advantage over their normal or benign neighbors^3^,^4^,^9^. Currently, most known cancer driver events were initially defined *in vitro* experimentally or using computational approaches based on their perceived roles in key cell signaling pathways^3^,^9^. Less is known about their *in vivo* roles and their interactions in cancer patients^8^. In fact, far fewer novel driver genes have been identified and recognized than were earlier anticipated, and it is becoming clear that many types of solid tumors contain only a limited number of driver genes in a background of many passenger genes^9^. How these cancer drivers interact or evolve to promote tumor progression remains a mystery.

While efforts were first devoted to correlating cancer driver events to patient survival and to clinical and pathological parameters such as tumor size, tumor grade and lymph node metastasis^10^,^11^,^12^,^13^,^14^,^15^, little has been revealed as to how those complex evolving genomic events interact to drive the changes of clinical and pathological phenotypes *in vivo*. Histologic grade is one of the most important and clinically useful biomarkers in determining the prognosis of invasive breast cancer^16^,^17^,^18^. The widely used Nottingham histologic grade, developed by Bloom and Richardson^19^ and modified by Elston and Ellis^20^, was evaluated based on three principal histologic or microscopic criteria, specifically, tubule formation, nuclear pleomorphism and mitotic count. Each of these features was scored from 1 to 3 and a combined overall score was categorized into grade 1 (score 3, 4 and 5), 2 (score 6 and 7) or 3 (score 8 and 9) or low, intermediate or high histologic grade (Fig. 1, left panel). Histologic grade remains the most well-known independent and reliable marker for predicting breast cancer aggressiveness^18^. Many studies have demonstrated that the grading of histologic features is most tightly correlated with tumor cell proliferation as assessed by mitotic activity^21^,^22^,^23^. Since proliferation advantage is the defining feature of a cancer driver gene, we hypothesized that histologic grade may serve as a marker for studying the driving genetic abnormalities *in vivo* in invasive breast cancer.

In our previous study^24^, using TCGA whole genome cancer sequencing data, we characterized the global genomic abnormalities associated with those clinically important histopathologic features of invasive breast cancer including histologic grade. Our study showed that accumulation of genomic defects was associated with a higher histologic grade, larger tumor size and receptor negativity. Invasive ductal carcinomas (IDC) with different histologic grades demonstrated distinct genomic abnormalities. TP53 and PIK3CA gene mutations were correlated with a high or low histologic grade, respectively. In this study, we examined the prevalent genetic/genomic changes seen in invasive ductal carcinoma and correlated them to the IDC histologic grade. Our results suggest that histologic grade may serve as a useful phenotypic marker to measure the oncogenic effect of cancer driver genes and their interactions *in vivo*. Remarkably, we discovered that different subtypes of genetic aberrations within a single cancer driver gene, such as PIK3CA or GATA3, are associated with opposite histologic changes in invasive breast cancer.

## Results

### Characteristics of the TCGA invasive ductal carcinoma cohort

**Figure 1:**
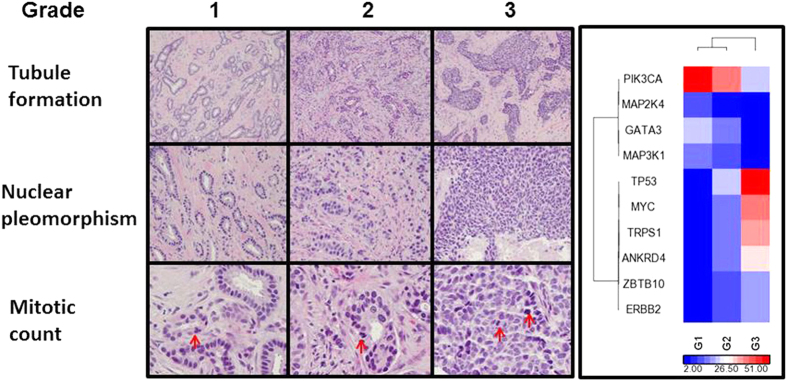
Invasive ductal carcinoma of various Nottingham histological grades associated with driver genomic abnormalities. Left. Representative microscopic images of invasive ductal carcinoma with various Nottingham histological grades. Red arrow indicates mitotic figure. Right. Hierarchical clustering of 10 candidate breast cancer driver genes correlated to the IDC histologic grades. This was performed using Pearson dissimilarity matrix for both genes and samples. The color indicates sample percentage (%) in G1, G2, and G3 as indicated by the scale.

The breast invasive carcinoma (TCGA, provisional) project has published whole genome sequencing data generated from 1104 tumor specimens collected from 1097 patients as of May 1, 2015. Each case also links to its corresponding surgical pathology report and digitized tumor slides. We reviewed all the pathology reports and collected the relevant clinical and pathologic data, including detailed histological grades (Supplemental Table 1). Among the 773 cases with a diagnosis of infiltrating or invasive ductal carcinoma, 680 (88%) have both genome sequencing data and overall histologic grade. Invasive ductal carcinomas of grade 1, 2 and 3 consist of approximately 9%, 40% and 51% of the TCGA IDC cases, respectively. A total of 1335 significantly mutated genes selected by MutSig (https://www.broadinstitute.org/cancer/cga/mutsig) and 62 significant copy number alterations (CNA) selected by GISTIC2 (www.broadinstitute.org/cancer/pub/GISTIC2/) were identified in these 680 IDC cases. TP53 and PIK3CA are the two most frequently mutated genes that were identified in 37% and 31% of the IDC cases, respectively. MYC amplification is the most frequently identified CNA (27%). Surprisingly, only five gene mutations (TP53, PIK3CA, GATA3, MAP3K1 and MAP2K4, Supplemental Table 2) and five amplified genes (MYC, TRPS1, ANKRD46, ZBTB10 and ERBB2, Supplemental Table 3) were found to be significantly associated with the IDC histologic grade. Nevertheless, this short list includes the most prevalent and well characterized breast cancer driver genes, TP53, PIK3CA and MYC. While the cases with one or more of these three genetic abnormalities accounted for 68% (461/680) of the TCGA IDC cases studied, this group of ten candidate cancer driver genes has successfully separated the high grade IDC from lower grade ones by hierarchical clustering (Fig. 1, right panel).

### Association of IDC histologic grade with prevalent driver genetic abnormalities

**Figure 2:**
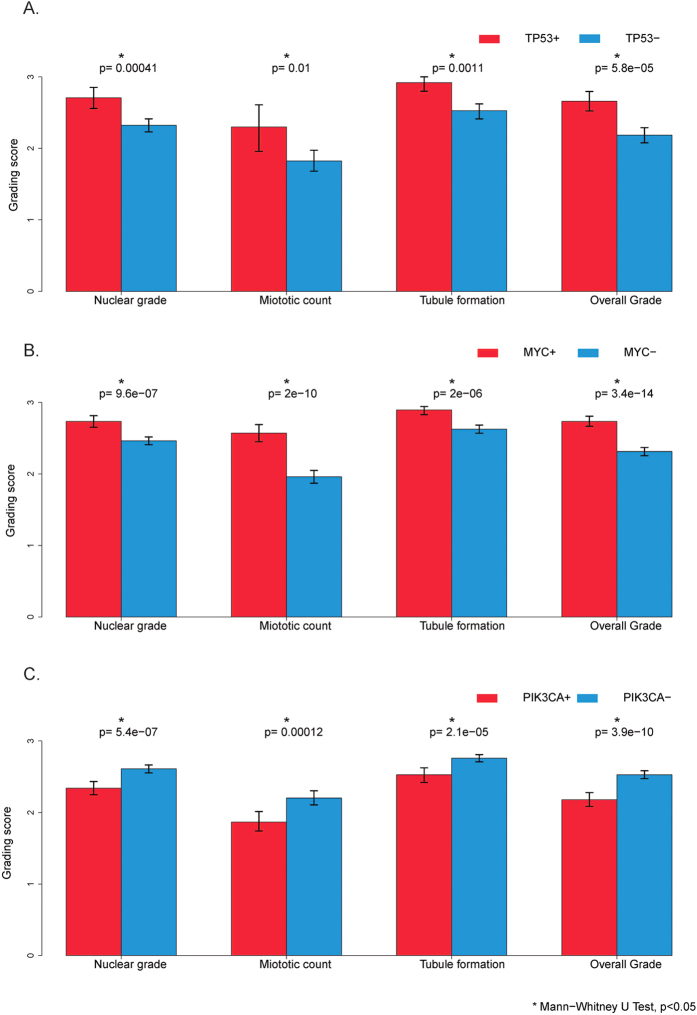
IDC histologic grade in correlation with different states of genetic abnormalities. (**A**) Tumors with a TP53 gene mutation, 254 cases; tumors without a TP53 gene mutation, 426 cases. (**B**) Tumors with MYC gene amplification, 177 cases; tumors without MYC gene amplification, 503 cases. (**C**) Tumors with PIK3CA gene mutation, 208 cases; tumors without PIK3CA gene mutation, 472 cases. *Indicates Mann-Whitney U test, *p* < 0.05.

Consistent with previous reports^2^,^11^,^24^,^25^,^26^, both TP53 mutation and MYC amplification were significantly associated with a higher histologic grade, and in contrast, PIK3CA mutation was associated with a lower histologic grade (Fig. 2A–C, *p* < 0.05). Similar correlations were also identified for all three histological grading features: tubule formation, nuclear pleomorphism and mitotic count, of the Nottingham histologic grading schema (Fig. 2A–C, *p* < 0.05). The tumors with a TP53 mutation and/or MYC amplification were seen in 49% of the TCGA IDC cases (334/680), and 9.8% (6/61), 30% (81/271) and 71% (247/348) of grade 1, 2 and 3 IDC, respectively (Supplemental Figure 1A). The IDCs with a PIK3CA mutation were seen in 30% of the TCGA IDCs (207/680), and 57% (35/61), 36% (98/271) and 21% (72/348) of grade 1, 2 and 3 of the TCGA IDCs (Supplemental Figure 1B), respectively. Among the PIK3CA mutated tumors, 39% (80/207) of them had a synchronous TP53 mutation and/or MYC amplification. These data suggest that histologic grade is closely correlated with specific driver genetic abnormalities and may serve as a phenotypic marker for studying these genetic or genomic abnormalities *in vivo*.

**Figure 3:**
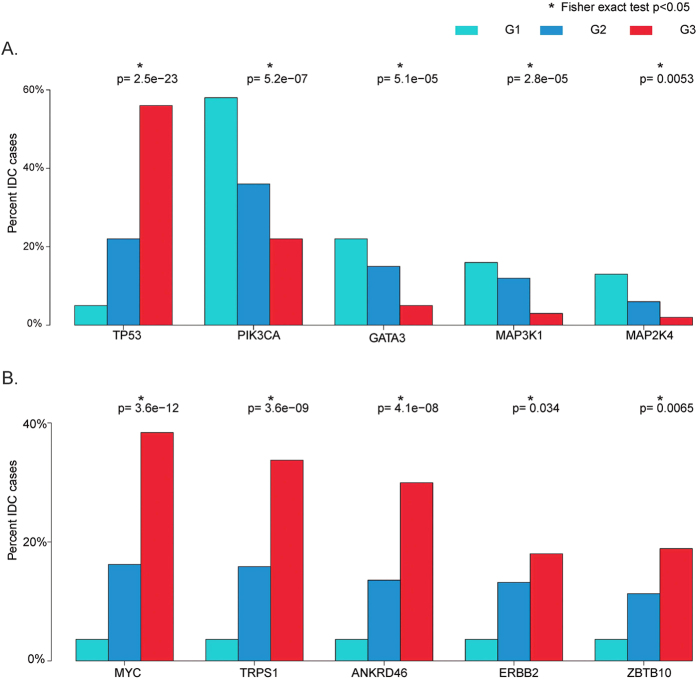
Significant mutated (**A**) or amplified (**B**) genes in correlation with histologic grade. *Indicates Fisher exact test, *p* < 0.05.

Only a small number of additional gene mutations and amplifications were identified to be significantly correlated with the IDC histologic grade. A TP53 mutation was the only gene mutation associated with a higher histologic grade. Interestingly, all other mutations associated with IDC histologic grade were correlated to a lower histologic grade, including the gene mutations in PIK3CA, GATA3, MAP3K1 and MAP2K4 (Fig. 3A). In contrast, all gene amplifications significantly associated with the IDC histologic grade were correlated to a higher histologic grade, including MYC and ERBB2 amplifications (Fig. 3B). This is thus the first observation that shows most gene mutations (except for TP53 mutation) are associated with a lower histologic tumor grade, while all gene amplifications are associated with a higher tumor grade.

### Effect of the driver genetic aberrations and their interactions on IDC histologic grade

**Figure 4:**
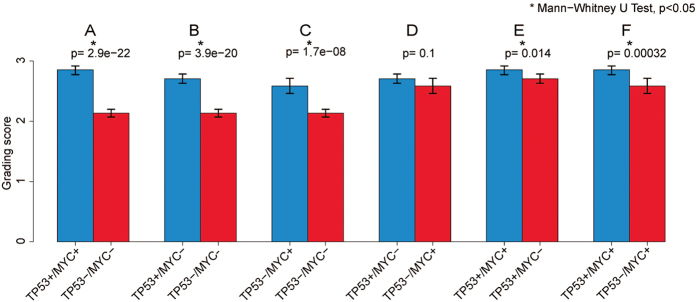
IDC histologic grade in correlation with different states of TP53 mutation and/or MYC amplification. IDC positive for both TP53 mutation and MYC amplification, 97 cases; IDC positive for TP53 mutation but negative for MYC amplification, 157 cases; IDC positive for MYC amplification but negative for TP53 mutation, 80 cases; IDC negative for both TP53 mutation and MYC amplification, 336 cases. *Indicates Mann-Whitney U test, *p* < 0.05.

A complex interaction at the genomic level among various genetic aberrations was observed in IDC as measured by histologic grade as a phenotypic marker. As reported previously^11^,^25^, TP53 mutated and/or MYC amplified IDCs showed a significantly higher histologic grade compared to IDCs without these genetic abnormalities (Fig. 4A, *p* < 0.05). TP53 mutated and MYC amplified IDC both were histologically at a higher grade (Fig. 4B,C, *p* < 0.05), and there is no significant difference in histologic grade between the IDC with only a TP53 mutation or a MYC amplification (Fig. 4D, *p* > 0.05). However, IDC with both TP53 mutation and MYC amplification showed a significantly higher histologic grading score when compared to those tumors with only a TP53 mutation or a MYC amplification (Fig. 4E,F, *p* < 0.05), suggesting a synergistic oncogenic effect by these two cancer driver genes.

**Figure 5:**
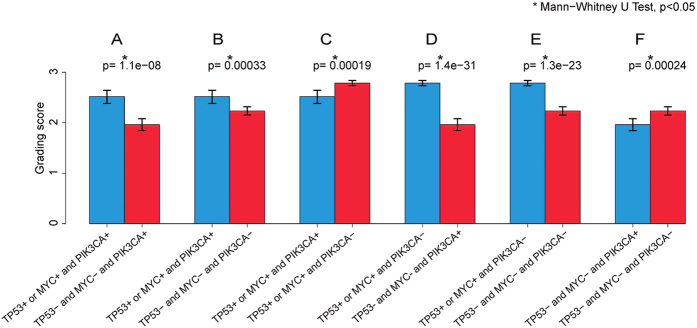
IDC histologic grade in correlation with different states of TP53 mutation, MYC amplification and/or PIK3CA mutation. IDC positive for TP53 mutation or MYC amplification and also PIK3CA mutation, 81 cases; IDC with TP53 mutation or MYC amplification but negative for PIK3CA mutation, 253 cases; IDC negative for TP53 mutation or MYC amplification but positive for PIK3CA mutation, 127 cases; IDC negative for TP53 mutation, MYC amplification, or PIK3CA mutation, 219 cases. *Indicates Mann-Whitney U test, *p* < 0.05.

**Figure 6:**
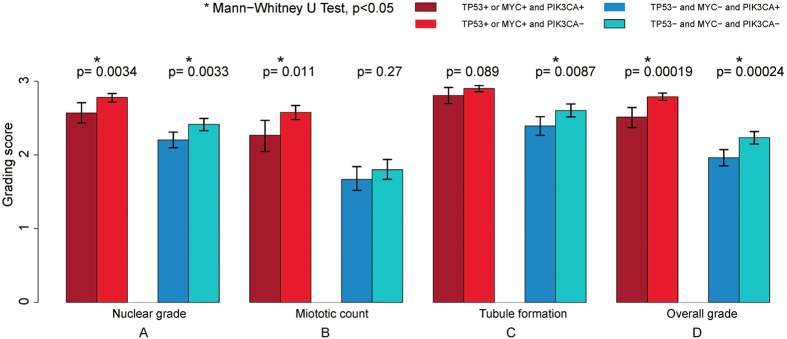
IDC histologic grading features in correlation with various states of TP53 mutation, MYC amplification and/or PIK3CA mutation. *Indicates Mann-Whitney U test, *p* < 0.05.

Although a PIK3CA mutation was found to be associated with a lower histologic grade (Fig. 2C, *p* < 0.05), the PIK3CA mutated IDC with a synchronous TP53 mutation and/or MYC amplification showed a significantly higher histologic grading score as compared to those without a synchronous TP53 mutation or MYC amplification (Fig. 5A,B, *p* < 0.05), suggesting that TP53 mutation and MYC amplification are more advantageous in tumor evolution towards a higher histologic grade or a more aggressive phenotype compared to a PIK3CA mutation. Interestingly, the TP53 mutated or MYC amplified IDC with a synchronous PIK3CA mutation showed a slightly lower, but statistically significant histologic grading score when compared to their counterparts negative for a PIK3CA mutation (Fig. 5C, *p* < 0.05). Further analysis of the three Nottingham histologic grading features correlated against the various states of the driver genetic aberrations showed that, in the TP53 mutated or MYC amplified IDC, the tumors with a synchronous PIK3CA mutation had a significantly lower mitotic count compared to their counterparts negative for a PIK3CA mutation (Fig. 6B, left panel, *p* < 0.05). In contrast, in the tumors without a TP53 mutation or MYC amplification, there is no significant difference in mitotic count between the IDCs with and without a PIK3CA mutation (Fig. 6B, right panel, *p* > 0.05). Interestingly, no statistically significant difference is observed in tubule formation, a tumor differentiation index, between the TP53 mutated or MYC amplified IDC with or without a synchronous PIK3CA mutation (Fig. 6C, left panel, *p* > 0.05). These results, for the first time, show that a synchronous PIK3CA mutation in the TP53 mutated or MYC amplified IDC may antagonize the oncogenic effect of the TP53 mutation or the MYC amplification by opposing the mitotic activity induced by them.

### Gene mutation and gene amplification within same cancer driver gene are associated with opposite IDC histologic changes

It is known that many cancer driver genes contain different types of genetic abnormalities. For example, PIK3CA and GATA3 genes may carry a mutation or amplification in invasive breast cancer. However, the functional roles for these diverse genetic changes during breast cancer development have not been well elucidated. We, therefore, compared the histologic grades among the IDCs with different types of genetic aberrations in all the significant cancer driver events we identified. It is interesting that various cancer driver genes have a quite distinct spectrum of genetic changes. For example, some genes such as MYC and ERBB2 show predominantly amplification and only rare other types of changes (Supplemental Figure 2). Interestingly, various genetic changes in the same gene may show quite different oncogenic effects as measured by histologic grade. For example, two main types of TP53 genetic aberrations are present in the TCGA IDC cohort, including 145 cases with a missense mutation and 103 cases with a truncating mutation. Our analysis shows that the TP53 truncating mutation appears more deleterious to TP53 gene function as evidenced by a significantly higher histologic grading score when compared to the IDC with a missense mutation (Supplemental Figure 3D, *p* < 0.05). The differential histologic effect of these two types of TP53 mutations are most likely attributed to nuclear pleomorphism (Supplemental Figure 3A, p = 0.011) and mitotic activity (Supplemental Figure 3B, p = 0.052). But more significantly, our study has discovered that gene mutation and gene amplification in two cancer driver genes, PIK3CA and GATA3, are correlated oppositely to the IDC histologic changes.

**Figure 7:**
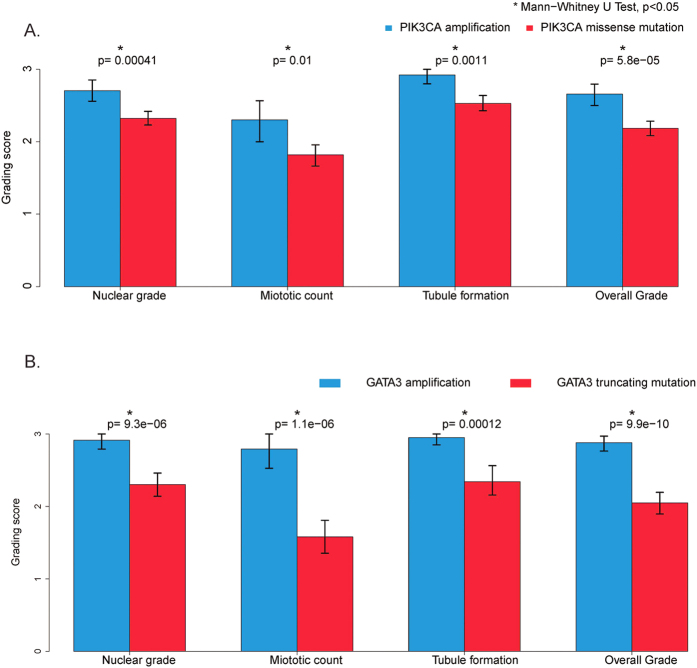
Histologic grade of IDC with amplification or mutation of PIK3CA (**A**) or GATA3 (**B**). (**A**) Tumors with PIK3CA gene mutation, 185 cases; tumors with PIK3CA gene amplification, 46 cases. *Indicates Mann-Whitney U test, *p* < 0.05. (**B**) Tumors with GATA3 gene mutation, 67 cases; tumors with GATA3 gene amplification, 37 cases. *Indicates Mann-Whitney U test, *p* < 0.05.

A PIK3CA mutation is a well-known genetic aberration identified across many types of cancer, including breast cancer^27^. Two main types of PIK3CA aberrations were present in the TCGA IDC cohort, including 185 cases with a missense mutation (27%) and 46 cases with a gene amplification (7%). Approximately one third (16/46) of PIK3CA amplified IDC (35%) also harbored a synchronous missense mutation. While PIK3CA mutations were known to be correlated with a lower histologic grade^12^,^13^, our study highlights the fact that IDCs with an amplified PIK3CA are predominantly of a histologically higher grade (35% grade II and 65% grade III), regardless of their PIK3CA mutation status. Further analysis showed that 80% (37/46) of these PIK3CA amplified IDCs had a synchronous TP53 mutation (32/46, 70%) or a MYC amplification (25/46, 54%). In contrast, in IDCs with only a PIK3CA mutation, only 37% (69/185) had a TP53 mutation (28%, 51/185) and/or a MYC amplification (18%, 33/185). While the PIK3CA mutation appears mutually exclusive to the TP53 mutation, a PIK3CA gene amplification often occurred concomitantly with a TP53 mutation or a MYC amplification. The co-presence of PIK3CA amplification with a TP53 mutation and/or MYC amplification in the same tumor may partially explain the high histologic grade states of those PIK3CA amplified IDC cases. However, all the remaining PIK3CA amplified IDC without a TP53 mutation or a MYC amplification were also at a higher histologic grade (4 grade II and 3 grade III) with no grade I tumors identified. Therefore, regardless of the presence of other cancer driver genetic abnormalities, the IDCs with a PIK3CA amplification showed a significantly higher histologic grade when compared to the tumors with a PIK3CA mutation (Fig. 7A, *p* < 0.05), suggesting that PIK3CA gene amplification may directly contribute to a higher histologic grade in IDC.

GATA3 is a transcriptional factor whose increased expression is associated with a better prognosis in patients with ER positive invasive breast cancer^28^. Two main types of GATA3 genetic aberrations were identified in the TCGA IDC cohort, including a gene mutation (67/680, 9.9%) or gene amplification (34/680, 5.0%). GATA3 gene mutations were predominantly in truncating forms with only three having a missense mutation and one having both a missense mutation and an amplification. Interestingly, the IDC with a GATA3 mutation and an amplification also had an “opposite” status in histologic grade. While a GATA3 mutation was mutually exclusive to a TP53 mutation or MYC amplification in IDC, the GATA3 gene amplification appeared clustered together with a TP53 gene mutation or MYC amplification. Only 22% (15/67) of the GATA3 mutated IDCs had a TP53 mutation (9%, 6/67) and/or a MYC amplification (18%, 12/67). In contrast, 85% (29/34) of GATA3 amplified tumors had a synchronous TP53 mutation (68%, 23/34) and/or a MYC amplification (47%, 16/34). As shown in Fig. 7B, the IDC with a GATA3 amplification showed a significant higher histologic grade compared to the tumors with a GATA3 mutation. In addition, the IDCs with a GATA3 mutation alone had a remarkably lower mitotic count compared to the tumors with a GATA3 amplification (Fig. 7B, *p* < 0.05). The remaining five IDC positive for a GATA3 amplification but negative for a TP53 mutation or MYC amplification were also of a higher histologic grade (two grade II and three grade III). In fact, almost half of those GATA3 mutated IDC at high histologic grade (8/18, 44%) also carried a synchronous TP53 mutation, a MYC amplification, or an ERBB2 amplification, which may partially explain the high histologic grade of these tumors.

## Discussion

While TCGA cancer sequencing project is near its completion, its data mining and knowledge translation remain an ongoing challenge^8^,^9^. One possible approach is to seek to find additional reliable and more efficient phenotypic biomarkers to directly correlate these complex genomic abnormalities with clinical outcomes. Although overall patient survival remains the ultimate biomarker to measure clinical success, it is not only time and effort consuming, but also is affected by many confounding factors such as tumor stage, patient immunity status and clinical treatment regimens. Here, we proposed to use the histologic grade as a phenotypic marker to compare different types of genetic aberrations and use them as a surrogate to decipher their functional interactions. The rationale for this includes Histologic: (1) grade has long been established as one of the most reliable and important prognostic indicators in the clinical management of invasive breast cancer^17^; (2) Proliferative advantage is the defining feature of a cancer driver event^9^, and the proliferative rate as measured by mitotic count is the most important component of the breast cancer histologic grading criteria^21^. Therefore, the histologic grade may optimally reflect the function of the cancer driving events *in vivo*; and (3) The histologic grade is part of a modern pathology diagnostic report of any invasive breast cancer case and most of the TCGA breast cancer cases already had this information embedded in their pathology reports. The corresponding digitized tumor slides were also available. We, therefore, tested the histologic grade as a phenotypic marker to study the driver genomic abnormalities and their interactions underlying invasive breast cancer. As expected, we confirmed that a TP53 mutation and/or a MYC amplification to be the major genetic aberrations associated with a higher histologic grade^2^,^11^,^25^ and the PIK3CA mutation to be associated with a lower histologic grade^26^. As expected, only a limited number of genes were significantly correlated with breast cancer histologic grade. Nevertheless, this small group of cancer driver genes was estimated to be responsible for the histologic grade in approximately 83% of TCGA IDC cases (Supplemental Figure 2).

The histologic grades of the remaining less than 20% of IDC are probably determined by additional, less prevalent or less frequently recognized cancer driver genetic abnormalities. One such example is the case of BRCA1 and BRCA2 gene mutations. BRCA1 and BRCA2 are two well-known breast cancer driver genes whose mutations are associated with a significantly increased breast cancer risk^29^. BRCA1/BRCA2 associated breast cancers are also known to be correlated with a higher histologic grade^30^,^31^. However, prevalence of BRCA1 and BRCA2 mutations is low in sporadic breast cancer patients^32^. The mutation/deletion rates of BRCA1/2 are 2.2% (15/680) and 3.2% (22/680), respectively, in TCGA invasive ductal carcinoma cohort. Probably due to its low prevalence, both BRCA1/2 mutations were not selected by the MutSig program to be significantly mutated genes. Both BRCA1/2 mutations also did not show a statistical significance among different grades of invasive ductal carcinoma although most of BRCA1/2 mutated TCGA IDC cases are histologically of higher grade (data not shown). While this may represent a pitfall in our effort to define cancer driver genes, identification of those low prevalent cancer drivers may need a larger number of IDC cases to determine their importance. Nevertheless, our study supports using the histologic grade as a phenotypic biomarker for the *in vivo* definition of a cancer driver event. Our findings are also supportive of the “Mountains and Hills” theory regarding the number of possible cancer driving genetic events that could be reasonably identified using current technology^9^. This theory proposes that the number of important cancer driver events (mountains) are limited and have probably already been identified, and the remaining less frequent cancer driver genes (hills) will need additional effort to be characterized^9^.

Driven by our hypothesis that histologic grade may be an ideal biomarker for *in vivo* definition of cancer driver events, we have further explored the *in vivo* interaction of those important cancer driver genes using histologic grade as a biomarker. As expected, TP53 mutation and MYC amplification play a similarly important oncogenic role and exhibit a synergistic effect in promoting a higher histologic grade phenotype. Surprisingly, we found that a PIK3CA mutation appeared to oppose the mitotic activity caused by a TP53 mutation or MYC amplification. Obviously, our observation argues against the view that a PIK3CA mutation acts as an activating oncogene as suggested by several *in vitro* studies^33^,^34^. However, our finding is in line with many recent clinical observations that a PIK3CA mutation is a marker of low grade tumor and thus a good prognosticator for invasive breast cancer^35^,^36^. One possible explanation for these seemingly contradictory findings is that a PIK3CA mutation may be an oncogenic driver event in early or low grade breast cancer development; while TP53 and MYC amplification are the oncogenic drivers that occur in a higher grade or a later stage breast cancer. It is possible that a PIK3CA mutation may act differently at cancer initiation than at progression. It may serve as an oncogenic driver gene during initiation to confer growth advantage over the surrounding normal epithelial cells but become clonally disadvantaged during progression when competing with other clones evolved to now display a TP53 mutation or MYC amplification, and thus functionally becomes a passenger gene. While the main consequence of a mutated or lost TP53 or a MYC amplification is uncontrolled cell proliferation, a defective cellular proliferation mechanism involving the PI3K-AKT pathway containing a mutated PIK3CA gene may not operate as efficiently as the pathway without such a mutation; therefore, leading to decreased mitotic activity in these tumors, as observed in this study. The co-presence of a TP53 mutation and/or MYC amplification in up to 40% of the PIK3CA mutated IDC in the TCGA IDC cohort also suggests that a high grade IDC could arise or evolve from a low grade lesion. Therefore, a tumor with both a PIK3CA mutation and a TP53 mutation or MYC amplification may represent a transitional phase of tumor progression from a low grade to a high grade tumor.

Recent TCGA genomic data analysis across a diverse group of 12 human cancer types revealed two top tumor classes with distinct types of genomic aberrations, the mutation (M) class and the altered copy number (C) class, existing in an inverse relationship, and representing two different levels of the oncogenic processes^37^. While M class tumors have different gene mutations, the C class tumors are grounded by TP53 gene inactivation and MYC driven proliferation with recurrent copy number changes. Surprisingly, we discovered that a mutation and an amplification of the PIK3CA gene or the GATA3 gene were correlated with opposite histologic changes in IDC. Mutations in both PIK3CA and GATA3 genes are virtually mutually exclusive for TP53 mutations or MYC amplifications. In contrast, amplifications in PIK3CA and GATA3 genes often present concomitantly with a TP53 mutation and/or a MYC amplification in the same tumor. Noticeably, these PIK3CA-amplified or GATA3-amplified IDCs are all at a higher histologic grade regardless of their states of TP53 mutation or MYC amplification, suggesting that the amplified PIK3CA or GATA3 genes may also be the driving events that promote the tumor to a higher histologic grade. Although the aforementioned M and C cancer classes are at the top of a global genomic pattern analysis across various cancer types, there appear similar M and C classes in invasive ductal carcinoma, in which M and C represent low and high grade IDC characterized by PIK3CA, GATA3 or other gene mutations and TP53 mutation, MYC amplification, or other gene amplifications, respectively. Our study demonstrated that the M and C class changes in an individual cancer driver gene, such as PIK3CA or GATA3, are correlated with opposite histologic changes. When both events occur within the same gene or same tumor, the effect of gene amplification will probably override that of gene mutation. Therefore, the IDCs with only a mutation in PIK3CA or GATA3 genes are histologically low grade or at early stage of cancer progression, but the amplifications of these genes are sufficient in and of themselves to drive the tumor to a high histologic grade, and therefore, represent a tumor progression event. In our study, all amplified driver genes are clustered together with a mutated TP53 and are correlated with a high histologic grade (Fig. 1, right panel). Therefore, it is possible that TP53 associated copy number genomic instability may be the cause of this tumor progression event^37^. While gene mutation at the nucleotide level may represent a localized genetic event, genomic instability associated gene amplification is often part of a genome wide, advanced genetic abnormality. Therefore, two breast tumors with mutation and amplification respectively in the same gene, such as in PIK3CA or GATA3, yield two distinct tumors of low and high grade, respectively. When both mutation and amplification occur in the same gene or in the same tumor, the effect of gene amplification will override that of gene mutation.

The above findings are important in improving our understanding of cancer progression and may have important clinical implication in designing approaches for personalized or precision oncology. Since an individual tumor often carries its unique set of driver genetic aberrations, treatment selection must consider the composition of the driver events and complexity of their interactions to avoid clonal selection by the targeted treatment^38^,^39^,^40^. For example, a low grade breast cancer contains only a PIK3CA mutation without other driver abnormalities may be a good candidate for PIK3CA inhibitor therapy. However, a higher grade breast cancer containing both a PIK3CA mutation and a TP53 mutation and/or a MYC amplification will not be the optimal candidate for the PIK3CA inhibitor only therapy. In this case, a combinatorial regimen including both a PIK3CA inhibitor and cytotoxic drugs might be a better option.

TCGA cancer genome sequencing project has provided an unprecedented opportunity for researchers to comprehensively characterize the genetic anatomy of almost all types of malignancies^41^,^42^. While this study aims at exploring the association of breast cancer histologic grade and those driver genetic changes and their interactions, it will be also important to further correlate or integrate our findings with other novel genome based molecular classifiers^43^,^44^,^45^,^46^,^47^,^48^. Such an effort may significantly improve our approach towards more precise or personalized breast cancer therapy.

## Materials and Methods

### TCGA Data

The Breast Invasive Carcinoma (TCGA, Provisional) dataset was accessed as described previously^24^. This data set, as of May 1, 2015, contains the experimental data encompassing gene mutations, copy number alterations (CNA), mRNA and protein expression and clinical data (patient survival, pathology reports and digitized tumor slides) from 1104 samples from 1097 patients with invasive breast cancer. According to histologic features, invasive breast cancer can be divided into two main types: (1) the special types include invasive lobular carcinoma, invasive mucinous carcinoma, and others; and (2) the invasive ductal carcinoma of non-specified type (IDC-NST), accounted for the majority (60–80%). Since the Nottingham histological grade was most suitable for grading the IDC-NST, only IDC cases were selected for this study. The pathology reports of all the TCGA breast cancer cases were reviewed by three pathologists (DS, ZP, TS) and the relevant clinical and pathological information were retrieved and summarized, including histologic type, grade, tumor size, lymph node status and ER, PR and HER2 status. The cases without overall histologic grade or gene sequencing data were excluded from this study. The final cohort used for this analysis includes 680 IDC cases. TCGA genomic data mining using cBioPortal (http://www.cbioportal.org/) and bioinformatics analysis using the Gene Pattern program (http://www.broadinstitute.org/cancer/software/genepattern/) were performed as described previously^24^,^49^,^50^.

### Selection of the genetic abnormalities correlated with breast cancer histologic grade

Fisher’s exact test was used for screening genes that were likely to be associated with the overall histologic grade. In the screening, we only considered genes with a mutation rate >1% or genes that were amplified and listed in Sanchez-Garcia *et al*. as important cancer genes^51^. The P-values given by the Fisher’s exact test were then adjusted to account for the multiple testing problem by using the “p.adjust” function in R with the “fdr” option. At the false discovery rate level 0.05, we identified 5 gene mutations (TP53, PIK3CA, GATA3, MAP3K1 and MAP2K4) and 5 gene amplifications (MYC, TRPS1, ANKRD46, ERBB2 and ZBTB10). The Mann-Whitney U test was used for testing if histologic grades were significantly different between the two groups of patients (e.g. TP53+ and TP53−). The Mann-Whitney U test was performed with the R function Wilcox.test. The confidence intervals were obtained by bootstrapping. More specifically, we resampled the samples with replacement 1000 times, calculated the mean grades of every resampled data, and set the 95% confidence interval as the 0.025^th^ quantile and 0.975^th^ quantile of the resampled mean grades.

## Additional Information

**How to cite this article**: Ping, Z. *et al*. A microscopic landscape of the invasive breast cancer genome. *Sci. Rep.* **6**, 27545; doi: 10.1038/srep27545 (2016).

## Supplementary Material

Supplementary Table 1

Supplementary Table 2

Supplementary Table 3

Supplementary Information
