# Supplementary material for: A microscopic landscape of the invasive breast cancer genome
Source: Sci Rep. 2016 Jun 10;6:27545. doi: 10.1038/srep27545 (PMC4901326; doi:10.1038/srep27545)

A microscopic landscape of the invasive breast cancer genome

Authors: Zheng Ping[1], MD, Yuchao Xia[5], MSc, Tiansheng Shen[1], MD, PhD, Vishwas Parekh[1], MD, Gene P. Siegal[1,4], MD, PhD, Isam-Eldin Eltoum[1,4] MD, Jianbo He[2], MD, Dongquan Chen[3], PhD, MD, Minghua Deng[5], PhD, Ruibin Xi[6], PhD, and Dejun Shen[1,4,*], MD, PhD

Affiliations: Department of Pathology, Division of Anatomic Pathology[1]; Department of Medicine, Division of Hematology/Oncology[2], and Preventive Medicine[3]; Comprehensive Cancer Center[4]; University of Alabama at Birmingham, Birmingham, Alabama 35249. Center for Quantitative Biology[5], School of Mathematical Sciences and Center for Statistics Science[6], Peking University, Beijing, China 100871

e-mail: dshen@uabmc.edu

Supplemental Table 1: Detailed histologic grade and significant genetic aberrations of IDC in TCGA invasive breast cancer cohort (provisional)

Blank space in the histologic grade indicates a missing score in the original pathology reports. The number “1” and “0” in the genetic aberration section represents either “positive” or “negative” for the genetic changes, respectively.

Supplemental Table 2: Significant mutated genes in correlation with histologic grade

The correlation of histologic grade and gene mutation states are examined by the Fisher’s exact test and then adjusted to account for the multiple testing problem. The mutations were ranked based on the p.adjust values. The significant mutated genes were highlighted in yellow. G represents histologic grade.

Supplemental Table 3: Significant amplified genes in correlation with histologic grade

The correlation of histologic grade and gene amplification states was examined by the Fisher’s exact test and then adjusted to account for the multiple testing problem. The gene amplifications were ranked based on the p.adjust values. The significant amplified genes were highlighted in yellow. G represents histologic grade.

Figure S1. Histologic grade distribution in IDCs with and without TP53 mutation or MYC amplification (A) and with and without PIK3CA mutation (B)

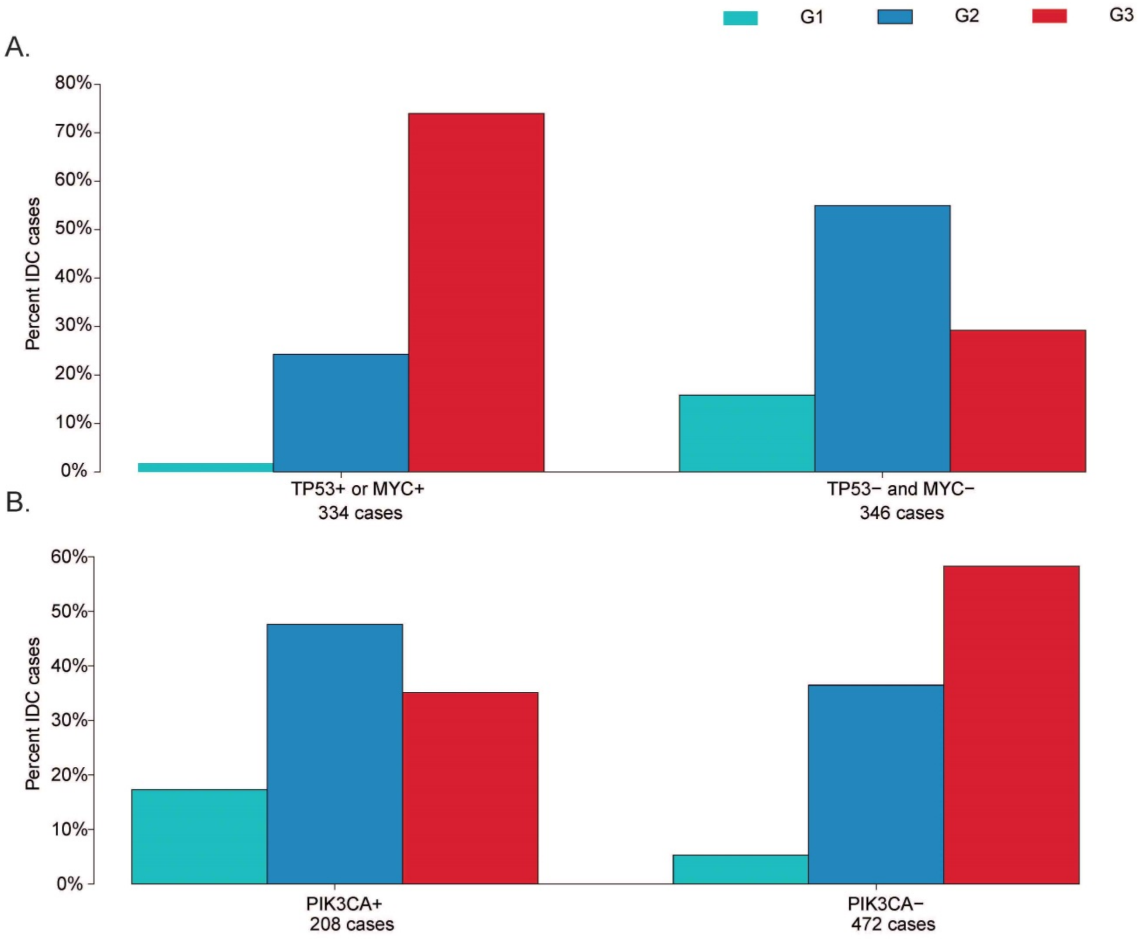

Figure S2. Distribution of different types of genetic abnormalities in TCGA IDC cohort

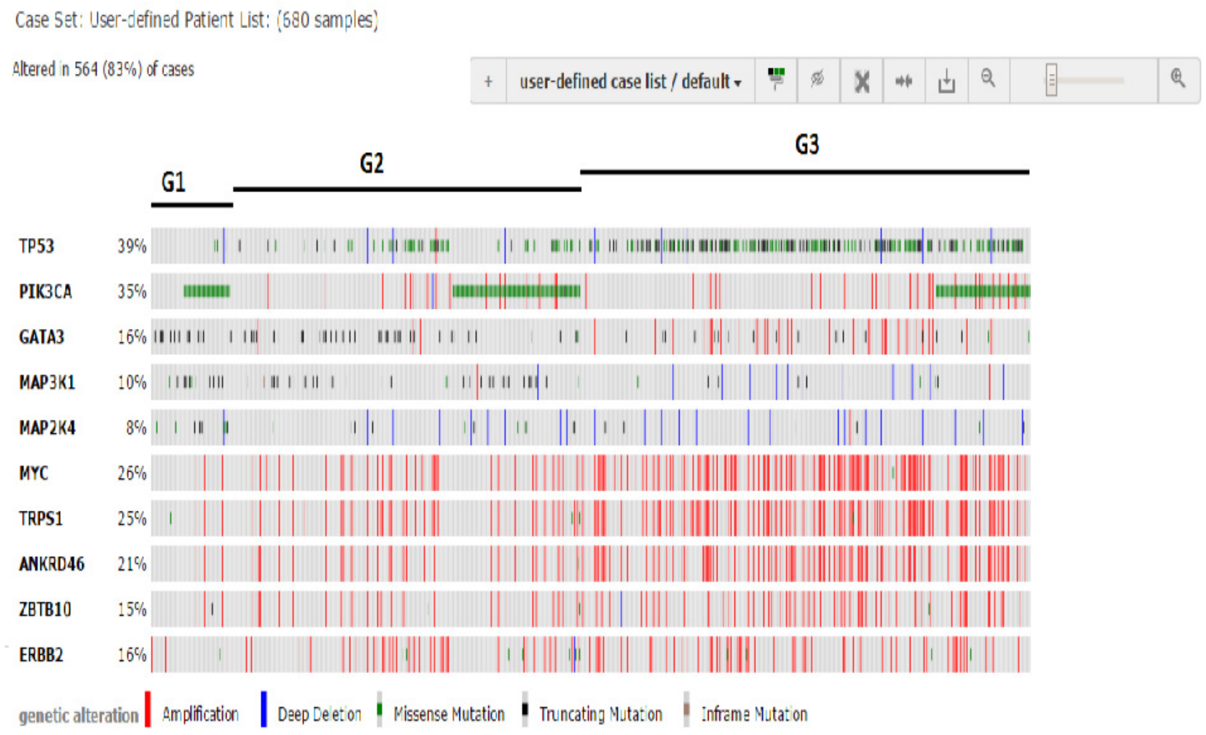

Oncoprint analysis was performed in 680 TCGA IDC cases at cBioportal website.

Figure S3. Histologic grade of IDCs with different types of TP53 gene mutations

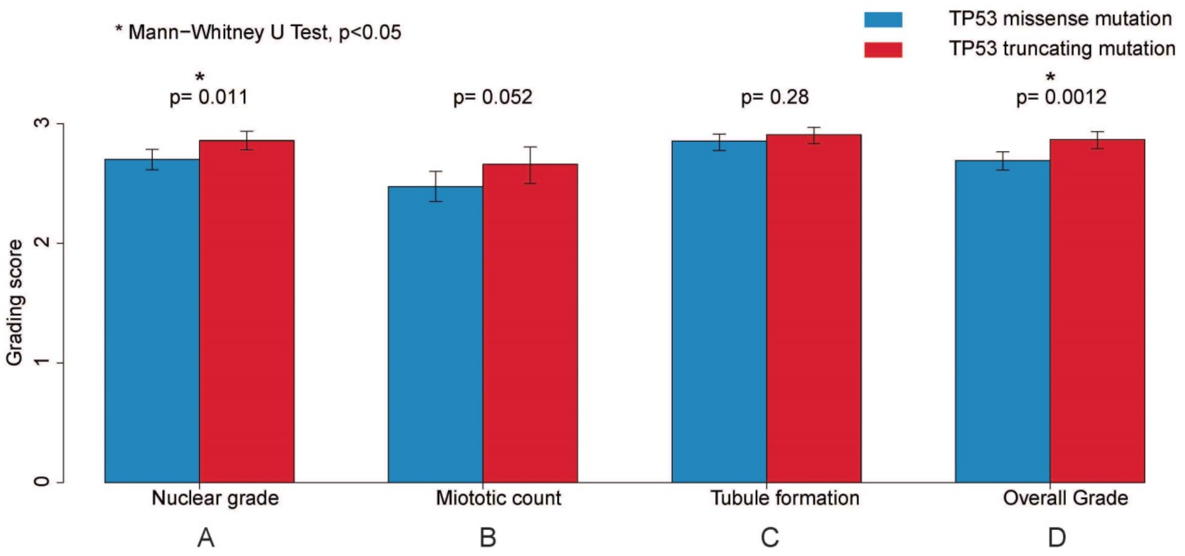

Supplement: Supplementary Information [file srep27545-s4.pdf]
